# The Neuropeptide System and Colorectal Cancer Liver Metastases: Mechanisms and Management

**DOI:** 10.3390/ijms21103494

**Published:** 2020-05-15

**Authors:** Aldona Kasprzak, Agnieszka Adamek

**Affiliations:** 1 Department of Histology and Embryology, University of Medical Sciences, Swiecicki Street 6, 60-781 Poznań, Poland; 2 Department of Infectious Diseases, Hepatology and Acquired Immunodeficiencies, University of Medical Sciences, Szwajcarska Street 3, 61-285 Poznań, Poland; agnieszkaadamek@ump.edu.pl

**Keywords:** colorectal cancer, liver metastasis, neuropeptides, nervous system-tumor interactions

## Abstract

Colorectal cancer (CRC), classified as the third most prevalent cancer worldwide, remains to be a clinical and research challenge. It is estimated that ~50% of CRC patients die from distant metastases, with treatment of this complication still posing significant difficulties. While liver metastasis (LM) cascade is known in the literature, its mechanisms are still unclear and remain studied in different research models. A connection is suggested between nervous system dysfunctions and a range of Neurotransmitters (Nts) (including Neuropeptides, NPs), Neurotrophins (Ntt) and their receptors (Rs) in CRC liver metastasis development. Studies on the role of NP/NP-Rs in the progression and metastasis of CRC, show the complexity of brain–tumor interactions, caused by their different forms of release to the extracellular environment (endocrine, autocrine, paracrine and neurocrine). Many stages of LM are connected to the activity of pro-inflammatory, e.g., Corticotropin-releasing Hormone Receptor 1 (CRHR1), Neuropeptide Y (NPY) and Neurotensin (NT), anti-inflammatory, e.g., Calcitonin Gene-related Peptide (CGRP), CRHR2 and Vasoactive Intestinal Polypeptide (VIP) or dual role neuropeptides, e.g., Substance P (SP). The regulation of the local immunological profile (e.g., CRH/CRHRs), dysfunctions of enteroprotective role of NPs on epithelial cells (e.g., NT/NT-R), as well as structural-functional changes in enteric nervous system innervation of the tumor are also important. More research is needed to understand the exact mechanisms of communication between the neurons and tumor cells. The knowledge on the mechanisms regulating tumor growth and different stages of metastasis, as well as effects of the action of a numerous group of Nts/NPs/Ntt as growth factors, have implications for future therapeutic strategies. To obtain the best treatment outcomes, it is important to use signaling pathways common for many NPs, as well to develop a range of broad-spectrum antagonists. This review aims to summarize the current knowledge on the importance of neuroactive molecules in the promotion of the invasion-metastasis cascade in CRC, as well as the improvements of clinical management of CRC liver metastasis.

## 1. Introduction

Colorectal cancer (CRC), classified as the third most prevalent cancer worldwide, after lung and breast/prostate cancer, is also the second leading cause of cancer-associated death [1,2]. Among all the cancers diagnosed in Europe, CRC is the second most widespread (after female breast cancer), also being the second most common cause of cancer-associated death (after lung cancer) [3]. Around 25% of CRC patients exhibit synchronous liver metastases (LM), with around 60% developing metachronous LM during the disease [4,5,6]. The liver is the most common metastasis location in CRC [7,8,9,10] and, most importantly, often the only metastasis-affected site [11]. LM in CRC, as a usually asymptomatic condition, is often recognized in late stages, shortening the survival time of patients by 5 to 20 months with no treatment [9,11,12]. The prevalence of LM is higher in stage IV of CRC cases than in curatively resected cases, being comparable to autopsy detected CRC (~85%) [13]. CRC remains to be a clinical and research challenge, as it is estimated that ~50% of CRC patients die from distant metastases, and treatment of this complication still poses significant difficulties [14,15,16].

While LM cascade is known in the literature [11,17,18], its mechanisms are still unclear and are studied in different research models [12,17,18,19,20,21,22]. LM in CRC is most commonly associated with anatomical location of organs and portal circulation (mechanical, hemodynamic or anatomic hypotheses) [9,13], but also with the location of the primary tumor (more commonly colon than rectum) and histological CRC subtype (most often adenocarcinoma) [7,23]. Liver metastases are also more often associated with commonly recognized CRC risk factors, such as sex (more prevalent in men), age (more common in women over 70 years of age, as compared to younger) [7], diabetes [24], high levels of low-density cholesterol (LDL) and LDL receptor (LDLR) [25] or alcohol consumption [26]. The role of obesity in CRC LM is still an open matter [27].

A strong connection is suggested between brain–gut axis dysfunctions, the expression of a range of Neurotransmitters (Nts) (including Neuropeptides, NPs), as well as Nerve Growth Factor (NGF) family of proteins called Neurotrophins (Ntt) and the progression of CRC (including distant metastases) [17,18,28,29,30].

The word “neuropeptide” was first used by David de Wied (1971), defining it as a peptide hormone devoid of the hormone of origin activity but able to exert effects on its own [31,32]. Other authors refer to NPs as small protein substances produced and released by neurons through the regulated secretory route, that lack endocrine activity in the brain but act on neural substrates to exert an effect on the target cell [33,34,35]. The concept of NPs was gradually redefined, recognizing that the name could also be used for regular peptide hormones, e.g., adrenocorticotropic hormone (ACTH), as they were also proven to be produced by neurons [31].

Neuropeptides are classified as complementary to classic Nts, sometimes even considered as their subtype, regardless of being produced by the cells of central and peripheral nervous system (neurons, glial cells), or non-neuronal cells (e.g., enteroendocrine cells, EECs), if they share the same genetic information, identical processes of synthesis and transport, as well as binding to similar families of receptors, allowing them to act on neural processes [34,35,36,37]. NPs are secreted to the synaptic space (neurotransmission), to tissue fluids (paracrine action) or blood (endocrine function) [35,36,38]. NP spectrum of activity is very wide, from the mentioned Nts to growth factors and key inflammatory mediators [39,40]. Recent studies indicate that NP signaling is evolutionarily conserved [41].

NPs are produced as biologically active precursor products of 100–200 amino acids (aa) and then trimmed to shorter peptides (3–100 aa), stored in neurons, in electron dense-core vesicles, together with one or two smaller Nts [36,39]. Currently, the human genome is described to contain around 90 genes coding NP precursors. There are ~100 peptides that can be produced and secreted by various neuron populations in mammalian brain [42].

NP action occurs through their specific, mostly G protein-coupled receptors (GPCRs), that are usually characterized by a seven-transmembrane region [33,34,36,38,39,40,43]. Understanding the structure of NP receptors (NP-Rs) opened the opportunity to design drugs which could be used in the treatment of diseases, such as inflammatory bowel diseases (IBD), irritable bowel syndrome (IBS), as well as epithelial-derived cancers, e.g., neuroendocrine tumors (NETs) or CRC [35,36,39,40,44,45,46].

Discovery of several NPs in the large intestine, as well as the elucidation of their roles, has a significant clinical application. Some of them (e.g., progastrin) were even considered as potential tissue CRC LM biomarkers [47]. Others (e.g., Glucagon-like Peptide (GLP) and peptide YY (PYY)) are used in immunophenotypic classification and prognosis of rectal NETs. It was proven that non-L-cell immunophenotype and larger tumor size (>1 cm) were associated with distant metastases [48]. Recent papers indicate that plasma concentration of neurotensin (NT), can, with high sensitivity and specificity, differentiate colon pathology (including CRC) from normal colonic epithelium [49].

The aim of this study was to summarize the current knowledge on the key roles of neuroactive molecules and their receptors in the human brain–gut axis, which have a proven or implied role in the mechanisms of CRC LM. CRC was chosen for the analysis, as in this cancer, LM is the most common cause of death, while the involvement of nerve dysfunctions accompanying CRC, as well as the role of the complex NP system in LM, still seems to be relatively poorly described.

## 2. Large Intestine Innervation as a Physiological NP Source

Innervation of the large intestine is a combination of central nervous system (CNS) elements (extrinsic innervation) with the enteric nervous system (ENS) (intrinsic innervation) [50,51,52]. Extrinsic innervation includes the autonomic nervous system (ANS) with sympathetic (SNS) and parasympathetic (PSN) branches, which originates directly in the CNS. The intrinsic innervation is composed of nerve cell clusters (enteric ganglia, submucosal (Meissner’s) and myenteric (Auerbach’s) plexuses (MPs)), and nerve fibers in the gastrointestinal tract (GIT) wall [50,52]. ENS nerve fibers innervate intestinal muscular layer, blood vessels and EECs, while the role of MPs in the large intestine is mostly the control of intestinal motility and descending phase of peristaltic reflex through the production of NPs, e.g., Gastrin-Releasing Peptide (GRP) [53,54], while Calcitonin-Gene-Related Peptide (CGRP) plays a cytoprotective role in intestinal mucosa cells [55]. In contrast, somatostatin (SM) inhibits intestinal motility and secretory activity [56]. The neuronal regulation via innervation of the crypts also concerns the differentiation and renewal of intestinal epithelial cells [51]. The production of NT Receptor 1 (NTSR1) and NTSR3 (but not NTSR2) was proven in sigmoid colon circular muscle and taenia coli. Functionally, this NP is responsible for direct (muscle) and indirect (neuronal/non-neuronal mechanism) smooth muscle contraction from ascending and sigmoid regions of the human colon, while the descending colon shows higher dependence on tachykinins (TAC), prostaglandins (PGEs), histamine and nitric oxide [57,58].

The main Nt of SNS in the proximal colon is norepinephrine (NE), acting through adrenergic GPCRs to control the vascular tone, enteric smooth muscle activity and inhibit the mucosal secretion [51,52].

PSN is represented by vagus nerve, which regulates the motility of the proximal colon. The main Nt of PNS is acetylcholine (ACh) associated with Muscarinic Receptors (MRs), also known as Cholinergic/Acetylcholine Receptors (CHRM) and nicotinic receptors [51,52]. The epithelial cell proliferation and differentiation are stimulated directly through the cholinergic myenteric neurons, through the release of ACh and serotonin (5-HT). In turn, myenteric neurons are regulated by axonal reflexes from sensory afferent neurons, which produce Substance P (SP) and CGRP [51]. Nerve fibers from the SNS and PSN innervate the whole large intestinal wall, creating synapses with ENS, with only the SNS fibers reaching the intestinal mucosa [52].

The structural-functional connection between the GIT and the nervous system also occurs due to the EECs (neuroendocrine, APUD cells), which are also a source of Nts and NPs. While 12 types of EECs are currently described, three types are present in the large intestine: enterochromaffin (EC, Ecm) cells (most prevalent), L cells (~14% of the EEC population in the rectum) and D cells (3–5% of the EEC population in all lower GIT) [59,60]. All of the NPs produced by EECs act through receptors on EECs, and other cells of the intestinal wall (e.g., smooth muscle and immune cells, enteric neurons) and nerve terminals. Some reports even suggest the presence of true synapses between neurons and EECs [52,60,61]. In the large intestine, 7 clear clusters of EECs can be differentiated, among which 4 clusters are EC/Ecm cells characterized by high expression of *Tph1*, encoding the Tryptophan 5-hydroxylase (TPH) enzyme for 5-HT synthesis [61]. Therefore, the major type of EECs in the large intestine are the EC/Ecm cells, producing 5-HT [60,61]. Further, two EECs clusters consist of L cells, rich in the *Gcg*, encoding GLP1, as well as seven clusters coding SM (D-cells). Among the L-cells, 4 sub-clusters can be distinguished, characterized by differential expression of *Gcg*, *Pyy* (PYY), *NTs* (NT), *Insl5* (Insulin-like Peptide 5, ILP5), *Cck* (Cholecystokinin, CCK) and *Sct* (Secretin). Changes in NP expression intensity were reported, dependent on location, cellular maturity (crypt-surface) and the anatomical region of the intestine (proximal-distal axes) [58,61]. Distal colonic/rectal L-cells also exhibit differential expression of the type-1A angiotensin II (ANG II) receptor gene (*Agtr1a)*, which causes a significant increase in plasma levels and in vivo production of GLP1, and PYY release in response to their stimulation with ANG II [61].

## 3. Alterations in Large Intestine Innervation during CRC

There is evidence of a direct link between the nervous system and cancer through synapses, non-synapse contacts, or humoral modulation, which contribute to two-way communication and influence cancer metastases. Similar to nerve structures, cancer cells produce Nts/NPs and their receptors [62,63]. In CRC patients, structural and functional changes of large intestine innervation can be observed. Interestingly, in the CRC LM, contrarily to the healthy liver, a lack of autonomic perivascular Protein Gene Product 9.5. (PGP9.5)- and Neuropeptide Y (NPY)-immunoreactive nerves can be observed [64].

### 3.1. Morphological Changes in Innervation and Neuropeptide Panel in CRC

Structural changes in CRC innervation mostly concern ENS, occurring most commonly in the form of gradual reduction, leading to the total destruction of the nerve structures [65,66,67]. Atrophy of submucosal and MPs within close proximity to the tumor occurs [67,68]. Among the NPs, a decrease in CGRP+ neurons and nerves was observed in both plexus types in the transitional zone between cancerous area and unchanged tissue. The decrease also concerned SP+ nerve fibers in all intramural plexuses [65] and NPY-ergic neurons, as well as the density of nerve fibers in both plexuses [66]. Interestingly, there were no significant quantitative differences in the numbers of SP+, SM+, Vasoactive Intestinal Polypeptide (VIP)-ergic and Pituitary Adenylate Cyclase-activating Peptide (PACAP)-ergic neurons, as well as SM+ nerve fibers in cancer, compared with healthy regions [65,66]. Lower numbers of VIP-ergic and PACAP-ergic nerve fibers were observed in submucosal and MPs than in control sections [66]. In turn, an unchanged density of galanin (Gal)-positive nerve fibers was observed, while the percentage of Gal+ neurons was higher in CRC (46%) than the healthy intestine (35%) [68]. A reduction in the size of Gal+ MPs in the vicinity of the tumor was also reported, as compared with unchanged tissue [67]. Mean Gal content in tumor was lower (9.38 ng/g) than in the morphologically unaltered intestine (12.27 ng/g) [68]. Ultrastructural changes in CRC patients include an increase in the mass of extracellular matrix (ECM), occurrence of myelin-like structures, numerous apoptotic cells, as well as the presence of mast and plasma cells in MPs in the tumor surrounding area [69].

### 3.2. The Perineural Invasion (PNI) in CRC

There are ongoing studies on the involvement of perineural invasion (PNI) of cancer cells in the modulation of tumorigenesis [62,63,70]. PNI might be an underestimated mode of metastasis spread, acting in combination with lymphatic and vascular invasion [70,71,72,73], as well as on its own [70]. In CRC, ~16–40% of the patients exhibited PNI characterized by neoplastic invasion of nerves, with altered molecular determinants of the process [70]. It is debated if nerve ablation can delay/inhibit the formation of tumors and/or reduce metastaticity [63].

The markers closely associated with PNI include Nts (e.g., ACh, NE and their receptors: AChR, NE-R), Ntt (NGF, Brain-Derived Neurotrophic Factor (BDNF), Glial Cell line-derived Neurotrophic Factor (GDNF) and their receptors: Neurotrophic Receptor Tropomyosin-related Kinase B (TrKB)), as well as typical NPs (e.g., SP, Gal, NPY/CGRP) [63,70]. PNI is a multistep process, during which a major role is played by the so-called perineural niche, together with numerous signaling molecules (including NPs/NP-Rs) [70]. There is a lack of detailed studies on the mechanisms of nerve–tumor interactions in PNI in CRC, as most of the research concerns different types of cancer (e.g., prostate and gastric cancers, pancreatic ductal adenocarcinoma) [63,70]. However, a prognostic role of PNI was proven in CRC. Defining PNI as a presence of cancer cells inside the nerve sheath, or at least 33% of the nerve periphery surrounded by cancer cells in CRC, shorter 5-year survival rates were observed compared with negative PNI. Additionally, positive correlations between PNI and lymph node metastases, tumor grade depth of invasion, clinical-stage, vessel invasion and tumor growth pattern were observed [74]. PNI was indicated as an independent bad prognostic factor in CRC [70,72,74], affecting both overall survival (OS) [74], cancer-specific survival (CSS) and disease-free survival (DFS) [72]. PNI is also an independent factor in CRC recurrence, points to a more malignant tumor phenotype and, as an important parameter, should be considered in pathological classification of CRC [70]. Recently, a large cohort study indicated that PNI is also more commonly observed in colitis-associated (90%) than in sporadic CRC [73].

### 3.3. Functional Innervation Disorders in CRC

Functional disorders in CRC and colitis concern mostly changes in interactions between large intestine innervation and the immune system [52,67]. Interestingly, such alterations occur on the level of NP-Rs, present on most of the immune cells. Anti-inflammatory roles of VIP and CGRP, as well as pro-inflammatory effects of serotonin and NPY, are also often underlined. In turn, SP has both anti- and pro-inflammatory effects. Apart from neurons, the production of Nts: ACh, choline acetyltransferase (ChAT), acetylcholinesterase, and both muscarinic/cholinergic and nicotinic ACh receptors, was also demonstrated on numerous immune cells (e.g., T and B cells, dendritic cells, macrophages), potentially extending the anti-inflammatory action of ACh in the large intestine [75].

Influence of some ANS Nts (e.g., NE, ACh) and their co-transmitters (e.g., NPY, adenosine triphosphate and/or VIP) on the proliferation of Intestinal Epithelial Stem Cells (IESCs) is also often underlined, despite little knowledge on the mechanisms of that process [52,76]. It seems that regulation of IESCs proliferation occurs with the participation of both branches of ANS, independently of ENS. Due to more numerous IESCs in the deeper regions of intestinal crypts, SNS and Nts can regulate the proliferation of these cells. ACh is also a PNS mediator, initiating a signaling cascade resulting in suppression of cyclin D1 and a downstream decrease in cell proliferation [76]. The role of ANS–IESC interactions is also considered in the context of differentiation of some kinds of colon cancers from somatic SCs, as well as maintenance of IESC-like properties under neoplastic conditions [76,77].

## 4. NPs and Their Mechanisms of Action in Pre-Cancerous Alterations and Colonic Inflammation

Up to 3% of CRC arise as a consequence of IBDs [78]. The source of many acute and chronic IBDs, caused by disorders of the brain–gut mucosa axis, are stress factors [50,52,58,79,80]. The main effects of stress on intestinal physiology are already the subject of many excellent reviews [62,80] and the participation of NPs, e.g., Corticotropin-releasing Hormone/Factor (CRH/CRF), NT, SP and VIP, in the pathogenesis of IBD is underlined [52,58,79,81,82,83,84].

The role of NTs/NPs in pathogenesis of inflammatory-associated CRC was also proven [52,62,75,84,85,86,87,88,89]. It needs to be noted that the action of NPs produced typically in the upper GIT portions (e.g., CGRP, NT, SP, VIP), studied in different models of colitis or on non-transformed colonocytes, can have both pro-inflammatory (NT, NPY, SP) and anti-inflammatory (CGRP, VIP) effects [52,89]. The role of fat–colonic mucosa interactions in IBD, with the participation of SP and its receptors: neurokinin 1 (NK-1R) and NK-2R, is described by some interesting research examples. An increase in production of mRNA of both SP receptors was observed in IBD mesenteric fat preadipocytes, while the levels of SP mRNA rose in ulcerative colitis (UC) preadipocytes. Moreover, the action of SP via NK-1R resulted in a release of interleukin (IL)-17 in Crohn’s disease (CD) and UC preadipocyte and IL-17R in IBD colon biopsies [83].

An especially important role in the development of acute intestinal inflammation is attributed to the neurotensinergic system (NT/NTSRs) [58,87,88,89,90,91,92]. Studies of the mechanism of NP action in colonic inflammation point out that both NT [87] and SP [86] mediate the acute phase of intestinal inflammation in vivo, inducing production of pro-inflammatory IL-8 cytokine by colonocytes. Furthermore, NT stimulates the expression in mesenteric fat depots [89]. An increase in NT/NTSR1 mRNA was also detected in the mesenteric fat of mice with chemically induced colitis [93]. The actions of SP and NT occur through a mechanism of phosphorylation and degradation of Nuclear Factor-kappaB (NF-κB), as well as phosphorylation of p65, with the participation of conventional Protein Kinase C (PKC) [87], or in the case of SP-isoform PKC delta [86]. It was also proven that the pro-inflammatory signaling pathway mediated by NT/NTSR1, with the participation of Matrix Metalloproteinase (MMP)-mediated release of Tumor Necrosis Factor (TNF)-α, transactivates Epidermal Growth Factor Receptor (EGFR), followed by the Mitogen-activated Protein Kinase (MAPK, MAP) pathway, increasing the expression of IL-8 in human non-transformed colonic epithelial NCM460 cells [90]. Studies on an animal model (rats) reported that NT, as a pro-inflammatory factor in the large intestine, also takes part in SP-dependent mast cell degranulation and is a key NP in the pathogenesis of *Clostridium difficile*-induced colonic inflammation [85,90]. Other research points to transactivation of the Insulin-like Growth Factor Receptor Type I (IGF1R) pathway by NT, resulting in combined effects of serine-threonine protein kinase AKT (AKT1) phosphorylation and NF-κB activation. These pathways participate both in pro-inflammatory and tissue repair signaling in colonic epithelial cells and can also play a role in IBD pathogenesis [94]. NT participates in the regulation of various enteric interactions with other systems, such as the CNS and neuroendocrine system. In intestinal carcinogenesis, the enterotrophic and enteroprotective effect of NT/NTSR system dysfunction on epithelial cells was also considered [89].

An important role in chronic stress and regulation of persistent inflammation, hence colon carcinogenesis, is played by the CRH (CRF) family of NPs. These group of peptides also includes urocortin I (Ucn1), II (Unc2), III (Unc3), CRH-binding protein (CRH-bp) and two distinct CRH receptors (CRHR1 and CRHR2), belonging to the GPCRs family [79,82,84]. Local expression of all of the CRH family components was observed in normal and inflamed colon and rectum. It was mostly localized in cells of MPs, submucosal ganglia, glial cells and nerve fibers, as well as non-neuronal components (e.g., smooth muscle cells, endothelial cells (ECs), mononuclear cells of lamina propria mucosae, and goblet cells in colonic epithelium). The role of the CRH family in the inflammatory process in vivo imitates changes resulting from stress exposure (e.g., colonic transit, motility, proto-oncogene Fos expression in myenteric neurons, visceral hypersensitivity and defecation), while treatment of in vitro cultured cells with CRF peptides confirms their participation mostly in secretomotor and mucosal alterations [79,84]. The mechanism of CRH family action is mostly based on the regulation of the local immunological profile [84]. Many authors point at the dual role of the CRH-CRHR signaling pathway in colon cancer tumorigenesis, progression and metastasis, with the role of both pro- and anti-inflammatory pathways [95,96,97,98]. CRHR2/Ucn2 signaling inhibits tumor growth and metastasis through downregulation of endogenous IL-6/IL-6R expression, reduction of STAT3 phosphorylation mediated by this cytokine, as well as inhibition of STAT3 in CRC cells, resulting in a block of expression of genes regulated by STAT3, as well as inhibition of cell cycle and EMT [96,98]. Additionally, the expression of Fas ligand in CRC cells was correlated with a loss of CRHR2 mRNA, poor tumor differentiation and high risk for distant metastases (including LM) [96]. CRHR2/Unc2 signaling turned out to also be a negative regulator of cell resistance to Fas/FasL-apoptosis in CRC via targeting the miR-7/YY1/Fas [98]. More importantly, activation of the CRH/CRHR1 most often leads to the promotion of inflammation, and upregulation of CRHR2—to an opposite response (anti-inflammatory). The pro-inflammatory action of the CRH system in the intestine occurs through a direct influence on immune cells (adaptive immunity), as well as cytokine production. The anti-inflammatory actions of CRH, Ucn1 and Ucn2 may also occur as a result of Toll-like Receptor 4 (TLR4) expression regulation, as is a part of the innate immunity [84].

## 5. Basic Steps of Colorectal Cancer Metastasis—Role of Neurotransmitters, Neuropeptides, and Neurotrophins

### 5.1. Proliferation, Migration, and Invasion of CRC cells

The first step in the invasion-metastasis cascade is the local invasion of tumor cells into the surrounding matrix, with the last step being the colonization of distant organs [21]. Uncontrolled proliferation, excessive migration and invasion of cancer cells are critical phenomena in the first step of CRC metastasis. Only a limited number of cancer cells can migrate to vital organs (e.g., liver, lungs and brain). Hence, the study of factors and mechanisms affecting the metastatic potential of these cells is also of great importance in relation to CRC LM [99].

#### 5.1.1. Neurotransmitters (Nts)

Among the classic Nts, the most studied for a potential role in the first step of CRC metastasis are: acetylcholine (ACh), norepinephrine (NE) and serotonin (5-hydroxytryptamine, 5-HT). The promigratory effect of NE on the migration of the human SW480 colon carcinoma cells (CCcs) was mediated by numerous β2-adrenoceptors (β2-AR) via tyrosine kinase activity [100]. In the case of ACh, increased proliferation of CCcs was mediated by transactivation of EGFRs, as well as through MRs (CHRM) [99,101,102]. Particular attention is paid to the role of the CHMR in CCc proliferation, survival, migration, invasion and metastasis. Detailed studies on H508 CCcs (expressing solely M3Rs) and SNU-C4 (expressing solely EGFR) showed that the mechanisms of this transactivation, resulting in increased proliferation of CCcs, are based on MMP7-catalyzed release of Heparin-binding EGF-like Growth Factor (HB-EGF). ACh-induced activation of EGFR and downstream Extracellular Signal-regulated Kinase (ERK) signaling also regulates transcriptional activation of MMP7 [101]. Other research reports that incubation of H508 cells with ACh resulted in a three-fold increase in cell migration, similar to that invoked by EGF action. Furthermore, ACh-induced HT-29 cell invasion was blocked by atropine [99]. Studies of CHRM agonist action in HT-29 and H508 cells, and in vivo models, proved their common functional interactions with CHRM through the stimulation of MMP1 expression, and deoxycholyltaurine-induced cell invasion [102]. Later research also indicated a role of ACh in post-M3R signaling pathways, increasing MMP1 expression and driving CCcs invasion. It was shown that ACh stimulated robust phosphorylation of p38 MAPK, which was EGFR-independent and could be blocked by PKC-α inhibition [103].

It has been reported that 5-HT is a key mediator of the growth of colon carcinoma allografts in vivo [104]. Studies on 5-HT receptor 3A (HTR3A) knockdown in the six CCc lines showed inhibition of their proliferation and colony formation, resulting in cell cycle arrest and the promotion of cell apoptosis [105]. Other studies confirm the role of serotonin and its receptors (5-HT(1B), 5-HT(3), 5-HT(4)) in enhancing CRC growth. Serotonin and its receptors’ agonists increase proliferation and growth of cells, and with the use of selective antagonists, inhibits proliferation and promotes HT-29 cells apoptosis [106,107]. In relation to mechanisms connected with CRC distant metastases, it was reported that tumor invasion occurs through the activation of the Axin1/β-catenin/MMP7 signaling pathway and 5-HT(1D)R. This receptor, by targeting Axin1 and dissociated β-catenin from the complex, activated β-catenin/Lymphoid Enhancer-binding Factor 1(LEF1)/T-cell Factor 4 (TCF4)/MMP7 signaling in CRC metastasis. Activation of 5-HT(1D)R directly induces cell invasion and migration both in vitro and in vivo, while its inhibition has a potent anti-metastatic effect via the Wnt pathway [108].

#### 5.1.2. Neuropeptides

##### Angiotensin II (ANG II)

In vitro research indicated promotion of growth, invasion and anti-apoptotic effect after the stimulation of CCc lines with ANG II (HT-29 and CT26) or angiotensinogen (ATG) (HT-29). It was also proven that hyperglycaemia induced ANG II activation in CCcs. Additionally, it was observed that a decrease in ATG production in the liver, mediated by cholesterol-conjugated antisense S-oligodeoxynucleotide, suppressed LM of HT-29 cells [24]. Other authors showed an increase in migration of other human CRC cells (DLD-1 and LIM2405) as a result of ANG II action via both receptor types (AT1R and AT2R) [109].

##### Endothelins (ETs)

Endothelins (ETs) include ET-1, ET-2, and ET-3, ET-A and ET-B receptors (ETAR and ETBR) (GPCRs family) with clearly defined functions, and upstream processing enzymes such as ET converting enzyme (ECE) [110]. ET-1 is an NP in the human colon with binding sites on neural plexuses and mucosa. In the 1990s, specific ET-1 binding sites were localized in the stromal tissues, including tumor vessels, fibroblasts and nerve fibers in human CRC. Hence, it has been suggested that ET-1 might play the roles of both mitogens and neurotransmitters through paracrine action [111]. For many years, ET-1 was investigated in the context of its impact on cancer progression (including CRC), as well as a potential antagonist of ET receptors in the therapy of various diseases [112,113]. A role of the ET/ETAR axis was also proven in the process of cancer spread and metastasis (including CRC LM) [112,114,115]. ET-1 production was detected in many human CCc lines [116]. In a rat LM model, a role of this NP in the promotion of tumor growth via ETAR was reported [117]. In vitro research (SW480 and SW620 cells) confirmed the role of ETAR in promotion of CRC LM. The effects of ET-1 on CCc survival, invasion and MMP-2 expression, occur through a phosphatidyl-inositol-3-kinase (PI3K)-mediated mechanism [115]. It has been recently demonstrated that enhanced proliferation, migration and survival of multiple CCcs via ET-1 also occurs through ETAR, in the mechanism of activation of YAP/TAZ, two transcription coactivators of the Hippo tumor suppressor signaling pathway [118].

High expression in the mouse colonic epithelial cells, both in healthy animals and those with experimental-induced colitis, also concerns ET-2. This NP was also observed in nerve fibers and MPs of the muscle layer, co-expressing with VIP [119]. The role of ET-2 and ET-3 in CRC progression is less recognized. However, it was reported that increased expression of both these peptides significantly weakens migration and invasion of human CCcs [110].

##### Galanin (Gal)

Gal/galanin receptor 1 (GalR1), as a novel receptor-ligand system that regulates CRC cell survival and drug resistance, was reported [120]. It was also suggested that at least a part of CRC cells with high Gal expression is more malignant and probably responsible for tumor recurrence. The positive correlation between high *Gal* expression and tumor metastasis, together with the aggressive behavior of CRC cells with high NP expression, might indicate the potential role of Gal in the spread of cancer stem cells (CSCs) in stage II CRC [121].

##### Gastrin/Progastrin

Progastrin (PG), gastrin and CCK act through the cholecystokinin-2 receptor (CCK2R, CCK-BR, CCK-B). Activation of CCK2R by gastrin stimulates a rapid tyrosine phosphorylation of the Focal Adhesion Kinase (FAK) pathway in CCcs (Colo320) [122]. Further studies confirmed the role of CCK2R in the regulation of invasiveness and motility of CRC cells [123]. The mouse research model also showed that autocrine/paracrine secretion of PG can promote proliferation of colonic epithelial cells indirectly due to stimulation of colonic myofibroblasts for production of IGF2 [124]. A pioneering study on the immature PG-derived peptide called Glycine-extended Gastrin (G17-Gly) reported that it can stimulate the invasiveness of CCcs. G17-Gly administration significantly enhanced the LoVo cells migration [125]. Other research isolated a novel splice variant of CCK-BR (CCK-BRi4sv) regulating intracellular free Ca^+2^ and CCcs proliferation though a gastrin-independent mechanism [126].

The potential role in CRC cell invasion and metastasis was also reported in studies on Colo320WT cells with mature G17. This peptide increased β-catenin expression [127] and activated the β-catenin/TCF-4 pathway, which leads to high expression of c-Myc and cyclin D1 [128]. Stimulation of HT-29 cells by G17 also caused an increase in phosphorylation of ERK1/ERK2 and AKT, increased Cyclooxygenase-2 (COX-2) expression, Prostaglandin E2 (PGE2) production and DNA synthesis, which resulted in cell growth [129]. Enhanced proliferation of colonic cells in vivo by non-amidated G17-Gly, as well as a second immature PG-derived peptide, C-terminal flanking peptide (CTFP), was confirmed in mice model of liver metastasis. However, CTFP does not seem to influence xenograft growth or the incidence of LM [130]. In turn, in the case of mouse colon cancer stem/progenitor cells in vitro, an increased proliferation through PG/G protein-coupled receptor 56 (GPR56) and PG/CCK2R systems was reported [131].

##### Neuromedins, Neuropeptide Y (NPY) and Substance P (SP)

Pro-proliferative effect in normal colon epithelial cells [132] and CCcs is exhibited by several NP/NP-R systems, e.g., GRP/GRPR [133,134,135] neuromedin B (NmB)/NMBR [136], NPY/NPY receptors (Y1, Y2, Y3, Y4, and Y5) [137] and SP/NK1R [138].

The family of neuromedins (Nms) consists of GRP, NmB and GRP18-27 (NmC) (bombesin-like peptides), NmK (neurokinin B), NmL (neurokinin A or neurotensin (NT)), NmN, NmS and NmU [135,139]. Three types of mammalian Nms (GRP, NmB and NmC) activate the bombesin receptors (BnRs) (GRPR (BB2), NmBR (BB1) and orphan receptor subtype 3 (BRS-3) (BB3)) [135,139,140]. An increase in HT-29 cell proliferation was obtained after 24 h of incubation with bombesin, GRP, NmB and NmC, due to their interaction with the GRP receptor [141]. The recent studies showed that overexpression of long noncoding RNAs (lncRNA), LINC01555 in CRC tissues, reinforced CCcs invasion through upregulating the expression of NmU [30]. The studies showed co-expression of NmB/NmBR, as well as autocrine action of this system in enhancing proliferation in normal (NCM-460 cells), as well as CCcs (Caco-2 and HT-29). Additionally, it was observed that NmB is 50–100% more effective in pro-proliferative activity on tumor cells compared to GRP [136]. Enhanced migration and proliferation of epithelial cells in rat intestinal cell lines-18 and Caco-2 monolayers is also caused by CGRP, acting via mast cells [55].

Normal human and mouse colonocytes do not express GRP/GRPR, but both components of this system exhibit differential expression in CRC and CCc lines [133,134,135,142]. GRP alone shows mitogen as well as morphogen activity, whereas the GRP/GRPR system regulates the histological progression of CRC in mice by promoting a well-differentiated phenotype [133]. The dependence of heterochromatin protein 1Hsβ expression on GRP/GRPR signaling was also shown. Inhibition of 1Hsβ caused increased invasiveness of human CCcs [143]. In vitro studies (Caco-2 and HT-29 cells) of the mechanisms of tumor cell metastasis showed that GRP promotes tumor cell motility and attachment to ECM, as a result of upregulation of Intercellular Adhesion Molecule-1 (ICAM-1) via FAK [144]. The mechanism increasing CRC migration via GRP/GRPR activity also includes Gα13-PRG-RhoA-ROCK, as well as Cox-2/PGE2 signaling pathways [145]. The nonamidated derivatives with pro-GRP C-terminus also manifested the pro-proliferative activity in the colonic mucosa in in vitro (DLD-1, HCT15, HCT116, HT-29), as well as in vivo conditions [146].

The Nm family also includes components of the neurotensinergic system (NT/NTSRs), the role of which in various cancer tumorigenesis (including CRC) is undisputed and reviewed [147,148,149,150,151]. The role of this system in CRC LM is less known. However, a role of the endocrine form of NT was described in the stimulation of growth of many CCc lines (SW480, SW620, HT-29, HCT116 and CI.19A), characterized with NTSR1 expression [152]. In HT-29 cells, the involvement of a complex of two structurally different receptors: NTR1 (GPCR family) and NTSR3 (type I receptor with a single transmembrane domain), was proven in the modulation of the MAPK pathway after NT stimulation and phosphoinositide (PI) turnover mediated by the NTSR1 [153]. Interestingly, it was proven in HT-29 and HCT116 cell lines that NT stimulates MAPK phosphorylation and cell growth through a pathway which does not involve EGF and IGFR systems [154]. Wang et al. showed that administration of sodium butyrate (NaBT), a Histone Deacetylase Inhibitor (HDACi), prevented NT-mediated induction of genes promoting cell proliferation and invasion (e.g., c-M*yc*, COX-2 and IL-8) [155]. Mouse model studies also showed promotion of tumor growth through stimulating action of NT/NTSR1 on the expression of miR-21 and miR-155 in colonocytes via AKT and NF-κB signaling [156]. Kim et al. showed differential expression of NT/NTSR1, lack of NTSR2 mRNA expression and consistent NTSR3 mRNA expression in all examined CCc lines, as well as promoter methylation of NTSR1/2. The role of NTSR1 in CCcs proliferation and migration was confirmed using an NTSR1 antagonist (SR48692) [157].

According to some reviews, the NT/NTSR1 system regulates different steps of CRC metastasis through three main signaling pathways (IP3/Ca^2+^/PKC/MAPKs, MMPs/EGFR/MAPKs (PI3K/AKT), or Rho-GTPases and non-receptor tyrosine kinase pathways [149]).

In relation to NPY, it was proven that the proliferation of intestinal epithelial cells is promoted via PI3K/β-catenin signaling and downregulation of miR-375-dependent apoptosis in these cells [137].

Almost two decades ago, it was reported that the proliferation of normal human colonic cells in response to SP is a result of NK1R activation via proinflammatory cytokines (IFN-γ, TNF-α and IL-1β). This effect was reversed by an NK1R antagonist (Spantide 1) [140]. Similarly, in human CCcs (LiM6 and DLD1), a block of NK1R using another antagonist (the clinical drug aprepitant) also caused significant growth inhibition. Blockage of the SP/NK1R signaling resulted in inhibition of canonical Wnt signaling [138].

##### Vasoactive Intestinal Polypeptide (VIP)/Pituitary Adenylate Cyclase-Activating Peptide (PACAP)

VIP/PACAP, together with their receptors (VPAC1, VPAC2/PAC1), can also promote growth and proliferation of normal and cancer cells [46,158,159,160]. Pro-proliferative effects of 5-day incubation with VIP, obtained from CRC patients, was observed on the Colo320DM cells [158]. The pro-proliferative effect of VIP in HT-29 cells was noted via the mechanism of induction of the cAMP-Rap1/Ras-B-Raf-ERK pathway [160].

A potential role in CRC growth has also been attributed to PACAP, which increases the number of viable cells and regulates FasR expression in HCT8 cells [159].

##### Corticotropin-Releasing Hormone/Factor (CRH/CRF)

A dual role of CRH/CRHR signaling was described in CRC and metastasis [96,97]. CRHR2/Ucn2 signaling inhibits CRC cell proliferation, migration, invasion and colony formation. Thus, CRHR2 downregulation is associated with a higher risk of distant metastases (including LM) [96]. By contrast, regarding CRH/CRHR1 signaling, a pro-inflammatory and, therefore, tumor-promoting effect, was observed in colitis-associated cancer [161]. Research on the detailed mechanisms of this process showed that CCc proliferation occurs through an IL-6/JAK2/STAT3-dependent mechanism and VEGF-induced tumor angiogenesis [97].

##### Glucagon (GCG) and Glucagon-Like Peptide 1 (GLP1) and GLP2

GCG promotes the proliferation of human and CRC cells in vitro and in vivo through binding to GCG receptor (GPCRs family). The downstream signals of GCGR include an activator of AMP-activated protein kinase (AMPK) and MAPK pathways, governing the development and progression of CRC [162].

GCG is cleaved into GLP1, GLP2 and other small peptides in intestinal L cells and brain neurons. Both of the peptides function in small intestine contractility and growth, while GLP2 stimulates mucosal enterocyte proliferation [60,163]. The use of GLP1 receptor (GLP1R) antagonist (exendin-4, Ex-4) resulted in a reduction of growth and survival in mouse CT26 CCcs via an increase of intracellular cAMP levels and inhibition of GSK3 and ERK1/2 signaling. Additionally, Ex-4 induced apoptosis, inhibited proliferation and caused changes in the morphology of cultured cells [164]. A weaker proliferation of the same mouse cells after Ex-4 application was confirmed by recent studies. However, in the human CCc lines (Colo320, Caco-2, HT-29, SW480 and LoVo), it was proven that this agonist did not enhance the proliferation and migration of these cells [165].

GLP2, as a main nutrient-responsive NP, functions in promoting cell proliferation and survival through CRC-related molecular pathways [166,167,168]. It was reported that GLP2 promotes an increase of mucosal colonic neoplasm in mice [169]. Activation of GLP2R directly stimulates protein synthesis by activation of the PI3K/AKT-mTOR pathway. GLP2 action on proliferation and growth of the intestinal epithelial cells appears to be indirect, being dependent on IGF1R signaling [166] and occurring through increased IGF1/2 expression in myofibroblasts [170].

##### Somatostatin (SM)

SM is produced by the CNS and PNS, EECs, inflammatory and immune cells, as well as many cancer cells. It has an inhibitory effect on cell motility and proliferation (G1 cell cycle arrest) and induces apoptosis [171,172,173]. SM acts through 5 receptor subtypes (sst1–sst5) (GPCR family) expressed by many normal and malignant cells [56,171]. In a study on Caco-2, HT-29 and HCT116 cells, expression of sst3/4/5, sst3/5 and sst2/3/5 respectively, was detected. The inhibitory effect of SM on the proliferation of CCcs was based on COX-2 downregulation via activation of two receptors: sst3 or sst5 [129]. Interesting studies on the interactions between SM-positive EECs and adjacent colonic SCs in crypt stem cell niche, indicated that the sub-population of aldehyde dehydrogenase (ALDH)-positive SCs is regulated by sst1 via a paracrine mechanism [174]. The impact of SM on uncontrolled CCcs proliferation and the potential role in CRC progression (including LM) can be explained by the genetic/epigenetic changes of the SM gene in CRC (which will be discussed later).

#### 5.1.3. Neurotrophins (Ntt)

Ntts include five structurally related growth factors: NGF, BDNF and neurotrophin 3, -4 and -5 (Nt-3, -4, and -5) [175,176,177]. This family of small proteins produced by neurons is associated with survival of sympathetic and sensory neurons, acting through TrKA, TrKB and TrKC (Receptor Tyrosine Kinases (RTK) family) and p75 neurotrophin receptor (p75NtR; TNF receptor family). The latter is often a downregulated tumor suppressor, as opposed to other commonly upregulated TrKs (and their ligands), acting as oncogenic factors [177]. Further, probable tumor suppressors also include TrKC [178] and the Nt-3 receptor (NtRK3) [179], with genetic and epigenetic changes causing their activation in CRC.

Numerous studies on the mechanisms of Ntt action in CRC progression (including the first step of metastasis) are focused on TrKB [176,180,181,182,183,184]. It was proven that TrKB mediates production of endogenous BDNF with some differences, depending on cell lines (WiDr, SW480, SW620 and Colo205), inducing proliferation and cell survival, as well as inhibiting apoptosis [180]. Ntt activate Ras, PI3K, phospholipase C-γ1 and MAPK pathways [175,176]. It was shown that both receptor types (TrKB and TrKC) induce cell growth and invasion, as well as function as anti-apoptotic factors [182]. Similarly, BDNF itself increased viability, migration, invasion and inhibited anoikis (detachment-induced apoptosis) in the CCcs [183].

**Figure 1:**
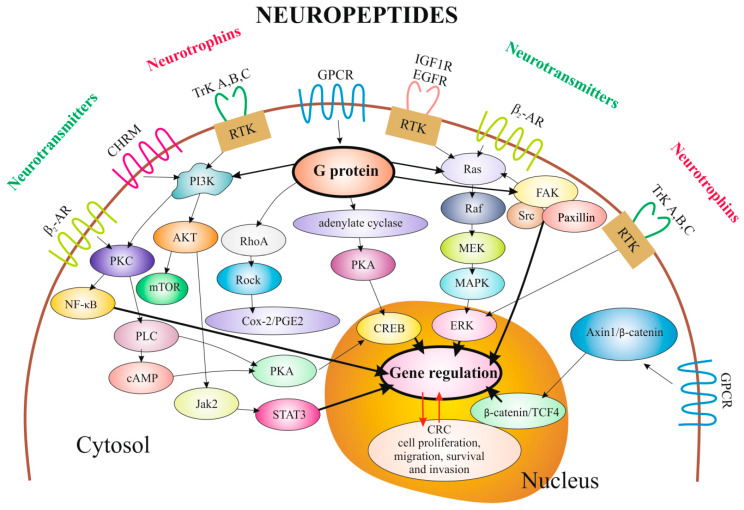
The schematic representation of the major cellular components and signaling pathways regulated by Neurotransmitters, Neuropeptides and Neurotrophins and their Receptors (β2-ARs, CHRMs, GPCRs, IGF1R/EGFR, TrKs), involved in a the first step of invasion-metastasis cascade in colorectal cancer cells, promoting CRC progression (including CRC LM). Abbreviations: Receptors: β2-AR-β_2_ adrenoreceptor; CHRM-Cholinergic/Acetylcholine Receptor; EGFR–Epidermal Growth Factor Receptor; GPCR-G Protein-coupled Receptor; IGF1R–Insulin-like Growth Factor Receptor type I; RTK–Receptor Tyrosine Kinase; TrK A, B, C-Tropomyosin-related Kinase A, B, C; Intracellular enzymes: AKT-Serine-threonine Protein Kinase (or PKB); Cox-2-Cyclooxygenase 2; CREB-cAMP response element-binding protein; FAK-Focal Adhesion Kinase; Jak2-Janus kinase 2; MAPK-Mitogen-activated Protein Kinase (called ERK); MEK-Mitogen-activated protein kinase kinase, a kinase enzyme which phosphorylates MAPK; mTOR–the mammalian Target of Rapamycin Kinase; NF-κβ-Nuclear factor kappa-light-chain-enhancer of activated B cells; PI3K-Phosphatidylinositol 3-kinase; PKA- Protein Kinase A, B (AKT), C; PLC-Phospholipase C; Ras–protein from small GTPase family; Raf-serine/threonine-specific protein kinases; RhoA-Ras homolog family member A; Rock-Rho-associated protein kinase; Src–kinase from non-RTK family; STAT3-Signal transducer and activator of transcription 3; *Others*: CRC–Colorectal Cancer; PGE2-Prostaglandin E2; TCF4–T-cell Factor 4.

The schematic diagrams of the main signaling pathways regulated by Nts/Nps/Ntt, as well as downstream signaling of their activated receptors, which are correlated with proliferation, cell cycle progression, migration and invasion of CRC cells, are shown in Figure 1.

In summary, the knowledge on the impact of selected Nts/NPs/Ntt on increased cell proliferation, migration and invasion of colon tumor cells is based on animal models, as well as in vitro studies using adequate assays and human (e.g., HT-29, H508, SNU-C4, DLD-1, LIM2405, SW480, SW620, Colo320, LoVo, HCT116, HCT8 and Colo205) or mouse (e.g., CT26) colon/colorectal adenocarcinoma cell lines with different metastatic potential. Most of the studies use HT-29 cells and their variants (WiDr, CI.19A), in which the presence of Nt/Np/Ntt receptors was confirmed. Most of the in vitro studies indicate a potential role of many neuroactive molecules in the metastatic process of CRC in vivo. Basic mechanisms of cell signal transduction involving Nts/NPs/Ntt were described, playing a major role in the regulation of genes important for CRC progression (including LM) (e.g., c-Myc, cyclin D1, COX-2, IL-8, MMPs, LEF1/TCF4)).

### 5.2. Colorectal Cancer Cell–Cell and Cell–Extracellular Matrix Loss of Adhesion

In this process, the participation of the NP/NP-Rs components upregulating MMPs production is especially important. The mammalian MMPs, particularly MMP1, degrade EMC and facilitate CRC cell invasion and metastasis. Their levels correlate with the clinical progression of CRC, hematogenous metastases and poor prognosis [102,185]. Production of MMPs (MMP1, MMP2 and MMP3) mediating cell migration was proven in LoVo cells after G-17Gly stimulation [125]. Interestingly, in tissue microarrays (TMA) of CRC LM, MMP1 and MMP2 were identified as consistently under-expressed, compared with primary CRC (pCRC). According to the authors, MMP1 levels in early CRC stages (II and III) were associated with an increased likelihood of distant metastasis, whereas rectal cancer in stage III recurrence was rather associated with MMP2 [186]. In turn, recent studies on serum MMP2 levels showed upregulation of this marker in CRC patients, as well as its correlation with clinical data (including lymph node and liver metastases) [187].

Increase in MMPs production (mainly MMP7 and MMP1) also upregulated expression of the Nts member ACh [100,102].

Furthermore, a study of CRHR2 signaling showed induction of changes in cell–cell junctions in two CRC cell lines (HT-29, SW620), affecting their ability to maintain cell–cell contact via the Src/ERK pathway. In HT-29 cells, cell adhesion remodelling, modification of cytoskeleton structures, as well as stimulation of migration and invasion ensued. All these phenomena can promote the metastatic potential of human CRC, resembling the EMT process [188]. In turn, other studies show lower expression of CRHR2 in CRC tissues and cell lines compared to control, suggesting contrasting effects of CRHR2/Unc2 signaling on tumor growth and EMT, with decreased expression of EMT-inducers and elevated levels of EMT-suppressors. In other words, downregulation of CRHR2 in CRC could be responsible for cell spread and be a factor of high metastasis risk [96,98].

Another NP-R with a potential role in this stage of LM is NTSR3 (Sortilin) and its soluble form (sNTSR3/Sortilin) [189]. The presence of Sortilin was proven at cell membranes of numerous cancer cells (including CRC cells) [190]. Specific binding of sNTSR3 and its internalization occurs in HT-29 cells, with a potential mechanism in this stage of metastasis based on activation of the FAK/Src-dependent PI3K pathway, accompanied with an increase in intracellular Ca^+2^ and a decrease of integrin mRNAs [189,191,192]. Hence, sNTSR3 action resulted in modification of desmosome structure, suggesting that these changes might lead to separation and spread of cells in early stages of carcinogenesis, easing metastasis [189,192].

Among neurotrophin receptors, it was shown that downregulation of TrKB increased anoikis sensitivity of CRC cells in vitro, with TrKB-induced anoikis suppression in CRC cells dependent on the PKB (AKT) signaling pathway. In other words, overexpression of TrKB (as an anti-anoikis molecule) could protect CRC cells from anoikis in the circulatory and lymphatic system [182].

### 5.3. Epithelial to Mesenchymal Transition (EMT) in CRC

EMT is an important process in CRC LM formation [21]. It was recently reported that the GPR56 (GPCR family) is significantly upregulated in some of the CCc lines (e.g., LoVo, DLD-1, SW480, HCT116), compared to control lines (NCM460). However, relatively low expression of GPR56 was detected in HT-29 cells. This peptide promotes CRC cell proliferation, migration and invasion, and is critical in CRC metastasis, due to EMT stimulation via activation of the PI3K/AKT signaling [15].

Another member of NP-Rs, acting on EMT, is ANG II Receptor Type 1 (AT1R). Use of specific AT1R and AT2R blockers caused inhibition of migration of human CRC cell lines (DLD-1 and LIM2405). In turn, ATR1 blocker caused an increase in E-cadherin expression and reduced Zinc finger E-box-binding homeobox 1 (ZEB1) and vimentin, while ATR2 inhibition lowered E-cadherin expression, without changing the levels of ZEB1 and vimentin [109].

Neurotrophin receptors, e.g., TrKB, also participate in EMT induction. In clinical samples, the inverse correlation was described between the expression of TrKB and E-cadherin. SW480 cell line studies confirmed the alleviation of malignant potential of these cells by TrKB knockdown. TrKB might play an important role in EMT and progression to metastasis [193].

### 5.4. Angiogenesis in CRC

A stimulating (e.g., epinephrine, NE), inhibiting (e.g., dopamine, SM), as well as dual role (stimulating or inhibiting) (e.g., NPY) of Nts and NPs on tumor angiogenesis has been described, which might suggest the role of these molecules in progression and metastasis of tumors (including CRC) [194].

Proangiogenic, as well as pro-inflammatory effects of NT, were demonstrated in an in vitro model (NCM460 cells overexpressing NTR1). These effects in acute colitis were mediated by NTR1-prolyl hydroxylase 2/HIF-1α-miR-210 signaling [92].

Two forms of gastrins (amidated G17 and G17-Gly peptides) were also indicated to increase expression of HB-EGF in Human Umbilical Vein ECs (HUVEC) and microvessel-derived ECs, as well as elevate the levels of MMP2, MMP3 and MMP9. Mean vessel density (MVD) in normal mucosa adjacent to CRC correlated with serum gastrin levels and HB-EGF expression in CRC patients [195]. Pro-angiogenic action of progastrin in CRC was also proven. Stimulation of cadherin phosphorylation in ECs, p125-FAK, paxillin and actin remodelling resulted in EC proliferation/migration, the ability of ECs to form capillary-like structures and enhanced permeability of endothelium [196].

Somatostatin is an endogenous inhibitor of both cell proliferation and angiogenesis. Interesting research using in vitro receptor autoradiography in submucosal and subserosal vessels localized near human CRC showed 3–5-fold overexpression of SM and SP receptors in the host veins within a close area (2 cm wide) surrounding human pCRC, as compared with veins located at a greater distance (5–10 cm) in control tissue. This finding suggests a regulatory mechanism presence on the levels of tumor vascular bed, which might be crucial for the development of CRC metastasis mechanisms [197].

Another pathway active during the Vascular Endothelial Growth Factor (VEGF)-induced tumor angiogenesis is the CRH/CRHR1 signaling, as one of the mechanisms in colitis-associated CRC [97,161].

A potential role of TrKB, positively regulating the expression of VEGF-A and VEGF-C, was also indicated in an in vitro model [180]. Increased secretion of VEGF-A in mouse rectal CMT93 cells is also caused, in a dose-dependent manner, by AT1R [198].

A list of Nts, NPs and Ntt and their receptors involved in the signaling pathways of the main steps of CRC metastasis (including distant metastasis) is presented in Table 1.

## 6. Role of Circulating Tumor Cells (CTC) in Liver Metastasis

CTC aggregates (up to 20 cells) from primary tumor sites might be the precursors of tumor metastasis (including LM) [199]. However, research on sensitive and specific CTC markers still poses numerous difficulties. Numbers of these cells in the blood of metastatic patients is insufficient for effective detection (<1 to <50 in 7.5 mL blood of a metastatic cancer patient), which motivates the search for more modern techniques of their detection, including other panels of cellular markers (apart from epithelial), typical for processes such as EMT, mesenchymal-epithelial transition (MET), with SCs traits, or in an immune evasive state [200,201]. NPs, being the subject of this review, have not yet been indicated as CTC markers.

Recent research shows that regulatory mechanisms of CTC-mediated tumor metastasis involve Tumor-Associated Macrophages (TAMs) by regulating the JAK2/STAT3/miR-506-3p/FoxQ1 axis. The study used different TAMs for evaluation of the associations of their sub-localization with EMT phenotype and ratio of mesenchymal CTC in CRC [202].

## 7. Tissue Expression of NP System Components in CRC and Liver Metastasis

CRC is a heterogeneous tumor, containing, apart from epithelial tumor cells, other cellular populations, e.g., cancer-associated fibroblasts (CAFs) [203], myofibroblasts [204], TAMs [202], B and T cells [205], tumor ECs (TECs) [206] and CRC CSCs [207], which might be a source of NPs and their receptors, playing a key role in CRC metastasis (including LM).

Cellular localization of most of the studied NPs concerns mainly epithelial tumor cells (TCs) [67,114,135,144,146,185,198,208,209,210,211,212], the “stromal” cells [117,208,211], TECs [117,208] and TAMs [95]. Expression was also described in typical EECs [213,214,215], neurons of submucosal and MPs [67], as well as cells of peritumoral veins surrounding human pCRC [194], or in blood vessels surrounding CRC [95].

A particular prognostic value in CRC is attributed to altered (especially elevated) expression of NPs and their receptors in tumor tissues, compared to control [95,121,135,198,210,212,214,216,217,218,219].

Peptides secreted in autocrine/paracrine signaling serve an important role in numerous signaling pathways, responsible for an increase in the concentration of cytosolic Ca^2+^ and proliferation stimulation [126,211], intensification of CRC migration/invasion [125], enhanced ECs activity in models of angiogenesis [196] or direct pro-carcinogenic effect [220]. Most of the studies of tissue expression, particularly of NP receptors, also suggest their usefulness in the context of CRC therapeutics.

From the studied group of NPs/NP-Rs, some were overexpressed in pCRC tissues compared with control, which might be linked to liver metastases. These molecules include (alphabetically): AT1R [198], BDNF [183], ET-1 [117], galanin [120], gastrin [210], NK-1R and SP [218], progastrin [47], TrKB [181], TrKC [182] and co-expression of BDNF/TrKB [183]. Lack of or significantly lower tumor expression versus control, which might also play a role in LM, was demonstrated for several NP/NP-Rs system components: CRHR2 [96], GRP/GRPR [142,146], SM [221], Sst2 [222], as well as Sst2 and Sst5 [223]. Some authors also observed elevated expression of NPs in pCRC, with lowered levels in metastatic liver (e.g., NmB and GRPR) [135].

Results of studies on the expression of renin-angiotensin system (RAS) components, conducted on murine models of CRC liver metastases, differ from those obtained from human CRC tissues [224,225]. In mouse metastatic liver, a lower expression of ATG and AT1R was observed, with elevated levels of angiotensin I converting enzyme (ACE) and ANG precursors 1–7 (MasR), compared to the surrounding liver. The treatment with captopril (inhibitor of ACE) in CRC metastases resulted in a decrease in LM volume and downregulation of ATG and AT1R expression, with increased ACE expression in the final stages of tumor growth [224]. Moreover, Wen et al. showed an immunomodulatory role of the RAS via liver Kupffer cells (KCs). Application of captopril increased the number of KCs in the LM in vivo and invasion in vitro, as well as appeared to alter the function of early, anti-tumor KCs during tumor progression [225]. In turn, Shimizu et al. showed that ANG II increases the expression of TGF-β1 in KCs. In other words, collagen build-up in metastasis area via the AT1a pathway was associated with resident KCs induction [226].

Studies on the role and mechanisms of action of Nt/NP/Ntt receptors in colon carcinogenesis and metastasis started almost three decades ago. They mostly concern NTSR [154,156,227], NmB-R [141,164] and TrK [180,181,182,183]. In the 1990s, expression of NTSR was detected in more than 40% of 19 CRC cell lines, with a lack of its presence in the normal colonic epithelium [228]. Furthermore, membrane localization of two structurally different NTSRs, namely NTSR1 (GPCR family) and NTSR3, as well as their internalization after NT stimulation, was described in HT-29 cells [154]. Higher expression of the NTSR1 gene in vivo was observed in colonic adenocarcinoma compared to adenomas. According to the authors, NTSR1 expression might be responsible not only for early stages of CRC development but also for its progression and aggressive forms, as successive NTSR1 tissue expression increase was described from colitis, through dysplasia, to CRC itself [227,229].

When it comes to the research on TrK tissue expression in CRC, the results are consistent for correlation of expression of these receptors with lymph node and peritoneal metastases, with less consistency when it comes to correlation with LM. In one study, only TrKC expression correlated with LM [182], while in another, this correlation was described for overexpression of TrKB [181]. In another, co-expression of both ligand-receptors, namely BDNF/TrKB, correlated with liver and peritoneal metastases [183]. Higher expression of ligands (BDNF) or TrKs correlated with clinical stage [180,182] and worse prognosis in CRC patients [181,183,193].

In the context of Sst1 to Sst5 expression in CRC and LM, the results vary. Loss of Ssst2 mRNA expression was described in Dukes’ stage D CRC and hepatic metastasis patients [222]. Other authors considered elevated Sst2 mRNA expression for a bad prognostic factor, as these patients exhibited shorter DFS [230]. Evangelou et al. described a negative correlation between the level of Sst2 and Sst5 protein expression in CRC with invasion and LM. In turn, the level of Sst2 expression was higher in lower-grade and rectum-located tumors, with patients with positive expression of both proteins (Sst2 or sst5) surviving longer [223].

## 8. Serum Levels of NP System Components in CRC and Liver Metastasis

Studies of serum concentrations of NP system components in CRC and/or CRC metastases (lymph nodes, liver), were mainly conducted in the context of their prognostic role or application in therapy. An increase in concentrations of Nts (e.g., serotonin) [231] and NPs were mainly observed in pCRC patients compared to control. This observation concerned the following NPs (alphabetically): ET-1 (and big ET-1) [117,208,209,232,233], galanin [67], gastrin/G-17 [234,235], GLP1/GLP2 [236], NT [49,237,238], PYY [236] and SP [239]. Serum VIP concentrations were also higher in metastatic CRC versus control [158].

ET-1 levels were also higher in LM patients compared to those without metastases [208], as well as patients with different types of LM (metachronous/synchronous) compared with control [232]. The systemic plasma levels of big ET-1 were higher in patients with CRC with Dukes’ D staging versus localized disease [209]. The levels of big ET-1 of > 4.2 pg/mL, age of patient >70 years and Dukes’ stage C, were indicated as factors of bad prognosis and independent prognostic values for OS [233]. However, other authors negate the prognostic value of serum ET-1 concentrations in CRC [232,240].

In the context of the role of gastrin/G-17 in CRC progression, the study results are also inconsistent. Some observed elevated concentrations of these peptides in CRC versus control [235,241], while others did not detect such differences [234]. However, significantly higher serum gastrin levels were demonstrated in patients with lymph node metastasis than patients without metastasis [234]. While gastrin concentrations of European CRC patients were higher in the tumor (>50 pg/mL) than in control, they were not indicated as a CRC risk factor and did not differentiate *Helicobacter pylori (Hp)* (+) and *Hp* (−) patients [241]. In precancerous lesions, some authors did not observe any correlation between hypergastrinemia and adenoma development [242], while others have shown that hypergastrinemia is a risk factor for colonic adenomas [243].

Elevated serum NT concentrations in CRC were indicated as one of the risk factors for colonic polyps or cancers (OR, 2.73; 95% CI, 1.33–5.59, *p* < 0.01) [238]. In turn, plasma serotonin levels were higher in patients with more severe TNM stages. High serotonin levels were shown to have a statistically independent prognostic value for poor OS and can be useful as a novel prognostic marker for CRC recurrence [231]. In a comparison of VIP concentrations in different metastatic GIT cancers (gastric, pancreatic and CRC), significantly higher levels of this peptide were detected in CRC in relation to both gastric [237] and pancreatic cancers [158]. The results collecting observations on tissue expression and plasma levels of NTs/NPs/Ntt in pCRC and metastatic CRC are shown in Table 2.

## 9. Genetic and Epigenetic Changes of Selected NPs and CRC Liver Metastasis

Profiles of many genes responsible for CRC development, and most probably associated CRC LM, were described. However, there is a lack of knowledge on LM-specific mutations that could be applied in everyday medical practice [245,246,247,248,249,250,251]. According to some authors, combinations of oncogenic changes are more important than specific events that determine the metastatic genotype of tumor cell early in carcinogenesis [245,248]. Although some of the papers comparing the genetic changes in pCRC and metastatic tumors, or advanced stages of CRC versus small tumors with less metastatic potential did not show significant changes in the Nt/NP/Ntt genes [246,247,249,251], there are some reports of such changes [245,250,252].

In the study of Koehler et al., among 23 up- and down-regulated transcripts in the high-stage and low-stage CRC group, upregulation of Ntt-3 precursor (BDNF, NGF2) was described [245]. Lim et al. defined 3 classes of gene mutations in CRC, assessing the presence or absence of mutations during LM development. Around 60% of changes were classified as Class 1 (shared between primary tumor and LM), which suggests the clonal origin of the primary tumor and LM. This research indicated 11 mutation-associated splicing events in the LM transcriptomes, including the splite-site *GPR56* mutation, which can result in a premature stop codon for all functional domains of *GPR56* (tumor-suppressor) and be responsible for CRC development. Although the analysis shows several interesting changes at the exome and transcriptome levels, the authors did not indicate significant LM-specific mutations [250]. Interestingly, among the differentially expressed mRNA (DEMs) in CRC, was a *GRP*, a significant increase of which could serve as an independent DFS prognostic gene [252].

The most common epigenetic alterations in CRC include aberrant methylation of DNA [178,179,253,254,255,256,257]. Hyper- or hypo-methylation might serve as an epigenetic biomarker for early detection, prognosis and response to chemotherapy in CRC [244,258,259]. The only, as of now, epigenetic marker approved by the US Food and Drug Administration (FDA) for CRC screening, is the methylation of the septin 9 (SEPT9) gene [259]. A search was also conducted for similar epigenetic biomarkers among the NP and Ntt genes. Therefore, e.g., expression of NT and NmN expressed in fetal colon, is reexpressed in ~25% of colon cancers. Differential expression of both these genes was also described in vitro—positive in human colon cancer KM12C cell line and negative in KM20, which was associated with epigenetic changes. Gene silencing in KM20 cells concerns methylation of the CpG sites in a distal consensus AP-1 site in the NT and NmN promoter. In turn, NT and NmN gene expression in KM12C was associated with demethylation of the CpG sites [253]. Methylation of the *NTSR1* promoter in some human CRC cells (KM12c, Caco2 and DLD1) and *NTSR2* in all six CRC cell lines (KM12c, Caco2, DLD1, HT-29, HCT116 and SW480) was also noted [157]. The NTSR1 gene is also often methylated in CRC in vivo, with the higher level of methylation occurring in laterally growing, large, non-invasive tumors, which is associated with better prognosis [260]. Other studies suggest that methylation-associated silencing of *NTSR1* is inversely correlated with invasiveness of CRC, and that a low level of methylation activates *NTSR1* and is responsible for malignant potential of CRC [257].

Studies of the SP precursor, TAC1, showed that high methylation levels of these markers in serum at 6-month follow-up, and SEPT9 at 1-year follow-up, were independent predictors for tumor recurrence and unfavorable CSS [261]. Furthermore, methylation inhibited the expression of the NTRK3 gene, observed in 60% of colon adenomas, and 67% of colon adenocarcinomas. Loss of these genes’ expression was associated with neoplastic transformation in vivo and in vitro [179].

Genetic/epigenetic changes also lead to activation of another potential proto-oncogene, TrKC. A decrease in TrKC expression was observed in a major portion of human CRCs. It was demonstrated that TrKC silencing by promoter methylation can limit tumor cell death. Additionally, the existence of two different tumor-associated TrKC mutants was proven in sporadic CRC. One of them is responsible for a gain-of-oncogenic function, while the other causes a loss of pro-apoptotic function, negating the tumor-suppressive effect of TrKC [178].

Other CRC-associated hypermethylation events, which significantly differentiated CRC and non-neoplastic tissue, as well as adenomas versus control, affected the *GLP1R* [254]. Studying the methylation levels of *NPY*, proenkephalin (*PENK)* and Wnt inhibitor factor 1 (*WIF1*), a potential diagnostic value of the combination of these three markers was proven in CRC [255]. In a study by Mitchell et al., the NPY gene was among the 7 others methylated in >50% of CRC samples, compared with a low level of methylation in non-neoplastic colorectal tissue [256].

Identification of genes prognostic for CRC with the use of bioinformatic methods showed that low expression of 4 out of 10 of them, including *GCG,* is associated with an unfavorable prognosis [262]. Similarly, recent studies confirmed that *GCG* is among the top five downregulated genes in CRC [263,264]. However, this gene is not significantly associated with distant metastases (including LM).

Other studied epigenetic alterations concern ET-2 and ET-3. It was proven that epigenetic inactivation through hypermethylation of *EDN2* and *EDN3* is common in both rat and human colon cancers [110].

The candidate methylation targets in pCRC included SM (88%) and the TAC1 (47%) genes. The degree of methylation was, in both cases, associated with a decrease in mRNA production. The intensity of methylation in the case of *TAC1* was higher in Dukes A/B than C/D. Hence, it cannot be associated with tumor progression (including LM). Significantly higher methylation of the SM was observed in low-level microsatellite stability (MSI-L) than non-MSI-L CRC [265]. In later publications on the SM gene, methylation status analyses showed a significantly higher level of promoter hypermethylation in CRC than in healthy young individuals, which can be linked to decreased SM production in CRC and uncontrolled cellular proliferation [221]. A significant correlation was found between serum methylation levels of *SM (mSM)* and CSS, which allowed to indicate mSM as an independent predictor for poor CSS. Patients with high serum mSM were noted to have a higher risk for cancer-specific death (HR = 1.96, 95% CI: 1.06–3.62) [266]. The molecular mechanisms of SM gene promoter methylation in CRC development and progression (including distant metastasis) are unknown. Common and significant DNA methylation was also observed in three other genes, among them, sst2, in CRC tissues compared to adjacent normal tissues [258].

It was also reported that hypomethylation of genes, e.g., ACE, might play a prognostic role in CRC. This process is important in enhancing of cell proliferation, colony formation, inhibition of apoptosis and is related to CRC prognosis [244].

Furthermore, regarding the CRHR2 gene, a recent study indicates higher levels of hypermethylation of the gene in patients with z colitis-associated CRC versus non-tumorous mucosa, which could be used in screening, prognostics, as well as evaluation of therapy outcomes in patients with UC-associated CRC [267]. These studies could also determine that hypermethylation of CRHR2 may be responsible (similarly as in the case of SM) for lowered tissue expression of this protein [96], but also cell spreading, being a high metastasis risk factor in CRC [98].

## 10. Targeting NP/NP-R System in CRC Liver Metastasis Molecular Therapy

Based on the knowledge of NPs/NP-Rs, attempts were made to introduce several drugs for patients with advanced stages of CRC, such as the label phase I/II study with G17-DT (Gastrimmune), with no observed tumor regression [268]. Falciani et al. applied the tetra-branched NT armed with 5-fluorouridine (5-FdU) and showed a 50% reduction in tumor growth as compared to animals treated with placebo [269]. In in vitro conditions, superior drug internalization of DOPC-NT_4_Lys(C_18_)_2_ liposomes with NT8-13 fragments and containing Doxorubicin (peptide-functionalized liposomes) was proven over the “pure” DOPC liposomes [270]. When it comes to NTSR1, the use of radioligand therapy with NTSR1-targeting agent, ^177^Lu-3BP-227, inhibited tumor growth and decreased its volume by 55–88% [271]. Effectiveness of VIP hybrid antagonist neurotensin(6–11)VIP(7–28) was also proven, both in vitro and in an animal model (rat), in inhibition of tumor growth and decrease of its volume or staging [272]. Based on the knowledge about BBS/GRP, using an antagonist of these peptides, RC-3940-II, the volume of HT-29, HCT-116 and HCT-15 tumors xenografted into athymic nude mice decreased by 25% to 67%. A better effect was obtained in combined therapy with RC-3940-II, 5-Fluorouracil (5-FU) and irinotecan [273].

If LM arises during the progression of CRC, a standard procedure dictates liver resection [14,16]. Proper and rapid regeneration of the liver after surgical LM removal has importance equal to the initial outcome of surgery [274]. Apart from hepatectomy, a combination of chemotherapy and targeted drugs, e.g., anti-VEGF and anti-EGFR monoclonal antibodies, are also applied [14]. A search continues for other forms of LM therapy, e.g., thermal ablation with hepatectomy, which could lower patient mortality and help to avoid two-stage hepatectomy [275].

In the context of this review, there are also attempts to apply the knowledge about the NP/NP-R system in the development of LM therapy. As an example, inhibition of the RAS system was tested as a therapeutic approach [225,274]. A mouse model of partial hepatectomy showed that early liver regeneration (LR) is promoted by captopril (ACE inhibitor) via an increased number of HSCs and MMP9 levels. The results of this study could be valuable in ensuring enough liver regeneration and prevention of tumor recurrence after hepatectomy [274]. Research by Wen et al., conducted on an orthotopic murine model of LM, showed varying effects of RAS component action on the number of KCs in the liver, possibly leading to in LM progression. The use of captopril and ANG-(1-7) increased the numbers of KCs in the liver (but not in the metastatic tumor), with the former reducing LM growth [225].

Of patients with different models of liver metastatic CRC, therapeutic approaches using neuropeptides (or their agonists/antagonists) were tested. However, their number was limited, including (chronologically): Sst2 [276], Gal, serotonin and octreotide [277], RAS [225,278,279] and NPY [280] (Table 3). Most of the used therapies inhibited tumor growth and liver metastasis formation. Among the treatments based on the knowledge that many NPs can act as growth factors or oncogenes, there are currently no drugs qualified for clinical trials in the context of CRC. However, some NPs, e.g., NPY methylated (mNPY) ctDNA, might serve a role of universal and easily applicable biomarkers in CRC patients treated with regorafenib. A high baseline level of mNPY ctDNA correlated with shorter patient survival, with the results potentially helping with treatment monitoring [280].

To prevent LM, a search is underway for inhibitors of its different stages, including CRC cell proliferation with NP-R participation using, for example, miRNAs. It was proven in in vitro and in vivo conditions that miR-148b inhibits cell proliferation and carcinogenesis via downregulation of the CCK2R gene on a translational level [281]. In studies on HT-29-bearing mice, significant inhibition of tumor growth by Nts dopamine was also observed, but only in combination with 5-FU. While dopamine did not exhibit a direct effect on tumor growth, it inhibited angiogenesis by decreasing TEC proliferation via VEGF-R2, MAPK and FAK kinase phosphorylation [282]. Polydopamine nanoparticles are also a promising immunotherapy candidate. Applied in tumor-bearing mice, they delayed tumor progression due to a sufficient amount of cytotoxic T lymphocytes (CTLs) and M1-type TAMs as well as the deficient number of immunosuppression-related cells in the tumor tissues [283]. Gao et al. demonstrated that CCKR-targeted immunotoxin (rCCK8PE38) also has potential as a new immunotherapy agent, decreasing tumor size in nude mice with HCT-8 tumor xenografts, as well as acting cytotoxically in two CRC cell lines [284].

## 11. The Main Headlines of the Review

In vitro and/or animal model studies on the mechanisms of Nts and NPs/Ntt action, confirm the participation of these molecule systems (mainly their overexpression) in main stages in CRC liver metastasis.

In the process of increased cell proliferation, migration and invasion (first step of metastasis), the most commonly described signaling pathways, activated by the studied neuroactive molecule systems, are Axin1/β-catenin/MMP7 (serotonin), cAMP-Rap1/Ras-B-Raf-ERK (VIP), EGFRs/PI3K/ERK/AKT/mTOR (ACh, serotonin, G17-Gly, GLP2), FAK (gastrin, progastrin, GRP), IP3/Ca^+2^/PKC/MAPKs (GCG, NT/NTSR1, neurotrophins), MMPs/EGFR/MAPKs (PI3K/AKT) (ACh, ET-1, NT/NTSR1), PI3K/β-catenin/TCF4 (G17, NPY), Rho-GTPases and non-receptor tyrosine kinase (NT/NTSR1), as well as Wnt signaling (SP). CRH/CRHR2 and SM/Sst3/5 systems exhibit inhibitory effects on the proliferation of CRC cells. Both in the cases of SM and CRHR2, epigenetic changes causing lowered production of these components were described in CRC patients, resulting in the ability for increased cell spread and appearance of distant metastases (including LM).

During cell–extracellular matrix loss of adhesion, the participation of, for example, ACh, CRHR2, G17-Gly, NTSR3 (sortilin) and TrKB, was confirmed. The main signaling pathways described in this step are AKT (TrKB), FAK/Src-PI3K/Ca^+2^ (NTSR3) and Src/ERK (CRHR2). The EMT program is induced by the action of, for example, AT2R, GPR56 and TrKB. During angiogenesis, mostly promoting (e.g., AT1R, CRHR1, G17, GlyG17, NT, progastrin, TrKB), but also inhibiting effects (e.g., SM) were described, as well as a dual role of NP/NP-Rs on the process (NPY).

In vivo studies confirm the production of numerous Nts/NP/Ntt and their receptors in neural structures (neurons, nerve fibers) or EECs, tumor microenvironment cells (TECs, TAMs, myofibroblasts), as well as in pCRC and metastatic cells. It is worth noting that peptide expression concerns different tumor cell populations, while the presence of their receptors is mostly characteristic for cancer cells. Liver metastasis presence was positively correlated with tissue expression/overexpression of many NP/NP-Rs (e.g., AT1R, BDNF, BDNF/TrKB, ET-1, gastrin, progastrin, SP/NK-1R system, TrKB and TrKC), as well as a lack or lower expression of some of them (e.g., AT2R, CRHR2, GRP/GRPR, Sst3 and Sst5).

Studies on the role of the serum NP/NP-R concentration were mostly conducted in recent years and indicate a diagnostic-prognostic role of these peptides. Higher levels of Nts/NPs were noted in pCRC versus control (serotonin, ET-1, big ET-1, galanin, G17, GLP1/GLP2, NT, PYY and SP), as well as in metastatic CRC than pCRC (gastrin, VIP), metastatic CRC versus control (ET-1) or in CRC compared to other types of GIT cancer (pancreatic, gastric cancers) (NT, VIP).

While there is a lack of description of liver metastasis typical mutations/epigenetic alterations concerning Nts/NPs/Ntt, a continuation of studies is essential in the elucidation of the mechanism of not only the development, but also metastasis, involving aberrant methylated DNA, or the discovery of a more universal epigenetic profile of CRC and LM.

## 12. Conclusions

1. Studies on the role of NP/NP-Rs in the promotion of the invasion-metastasis cascade in CRC, show the complexity of brain–large intestine–tumor interactions, caused by their different forms of release to the extracellular environment (endocrine, autocrine, paracrine and neurocrine). More research is needed to understand the exact mechanisms of neuronal-tumor cells communication.
2. Many steps of CRC promotion, progression and liver metastasis are connected to the activity of pro-inflammatory (CRHR1, NPY, NT), anti-inflammatory (CGRP, CRHR2, VIP) or dual role (SP) NPs, regulation of the local immunological profile (CRH/CRHRs), dysfunctions of the protective/enterotrophic role of NPs on epithelial cells (NT/NTSR system), structural-functional changes in ENS innervation of the large intestine in CRC (including PNI), or other tumor-promoting factors (bacterial GIT infections, such as *H. pylori*).
3. The knowledge on the mechanisms regulating tumor growth and different steps of metastasis, as well as effects of the action of a numerous group of Nts/NPs/Ntt as growth factors, have implications for future therapeutic strategies. For obtaining the best treatment outcomes, it is important to use signaling pathways common for many NPs, as well as the development of broad-spectrum antagonists.

## Figures and Tables

**Table 1:** Neurotransmitters (Nts), Neuropeptides (NPs) and Neurotrophins (Ntt) and their receptors playing a role in the progression of colorectal cancer (including CRC liver metastasis) and the major mechanisms of action of the neuroactive molecules in the most important steps of cancer metastasis.

Stage of Liver Metastasis	Type of Molecule/Receptor			Mechanisms/Signaling Pathways
	Nts	NPs	Ntt	
Cell proliferation, migration, survival	ACh NE Serotonin β2-AR CHRM	ET-1 Gastrin, PG, G17 CRH GCG GLP1/2 GRP/GRPR NT NPY SP VIP GPCR family	NGF BDNF Nt-3,-4-,-5 TrK A, B, C	See Figure 1
Cell-Cell weakening and CEM loss of adhesion	ACh	G17-Gly CRH/CRHR2 NT/sNTSR3	TrKB	↑MMP1, -2, -3, -7; Src/ERK; FAK/Src/PI3K; AKT
EMT	E NE	ANG/ATR1/ATR2 GPR56	TrKB	PI3K/AKT; E-cadherin, ZEB1, vimentin expression modulation
Angiogenesis		ANG/ATR1 CRH G17, G17-Gly, PG NPY, NT/NTSR1	TrKB	↑VEGF-A, VEGF-C; Cadherin phosphorylation; P125-FAK, paxillin, actin remodelling; ↑HB-EGF; ↑MMP2, -3, -9

Legend: ACh—acetylcholine; ANG—Angiotensin; AT1R/2R—Angiotensin 1/2 receptors; β2-AR—β_2_ adrenoreceptor; CRC—Colorectal Carcinoma; CRHR1/CRHR2—Corticotropin-releasing Hormone (Factor) Receptors; CHRM—Cholinergic/Acetylcholine Receptor; CEM—Cell-extracellular Matrix; E—Epinephrine; EMT—Epithelial-mesenchymal Transition; ET/AR/BR—Endothelin/Receptor A/B; FAK—Focal Adhesion Kinase; G—Gastrin; GCG—Glucagon; GLP1/2/R—Glucagon-Like Peptides/Receptor; GPCR—G Protein-coupled Receptor; GPR56—G protein-coupled receptor 56; GRP/R—Gastrin-Releasing Peptide/Receptor; HB-EGF—Heparin-binding EGF-like Growth Factor; MMPs—Metalloproteinases; NE—Norepinephrine; NPY—Neuropeptide Y; NT/NTSR3—Neurotensin/NT Receptor 3; PG—Progastrin; SP—Substance P; TrK A, B, C—Tropomyosin-related Kinase A, B, C; VEGF—Vascular Endothelial Growth Factor; VIP—Vasoactive Intestinal Peptide; ↑/↓—significantly increased/decreased.

**Table 2:** Tissue (T) expression, serum (S) levels of NPs and their receptors in CRC, with their potential roles in metachronous (m) or synchronous (s) liver metastasis (LM) [references].

Neuropeptides/NP Receptors			CRC	
Type	Source	Cellular localization	Primary	Liver Metastases
ACE	T		#, ↑ versus C [244]	
AT1R/AT2R	T	cell membranes (AT1R), cell nuclei (AT2R) of TCs [198]	♦, ↑ of AT1R; ♦↓ of AT2R [198]	
BBS, GRP, pro-GRP	T	TCs, including signet-ring cells [135,142,144,146,212]; EECs [214]	100% (+) pCRC [135]; ♣, (+) [144]; ♦, #, ↓ [142,146]; (+) [212]; 30% pCRC with intracellular changes [214]	Aberrantly expressed mRNA [135]; ND [144]
CCK	S		↓ versus C, ↑ CRC versus GC [237]	
CCK2R	T	TCs [123]; epithelial TCs (33%), non-epithelial cells (39%) [211]	44.4% (+), 26.7% (++) [123]; CCK-BRi4sv in 50% CRC and 100% polyps [126], 6/8 (+) [211]	
CRHR1, CRHR2 (CRF1, CRF2)	T	Epithelial TCs (CRF2) [188]	♦, #, ↓ mRNA/protein versus C [96]; ♣, ↑ Ucn2/3, CRF2 [188]; ND [217]	♦, #, ↓CRHR2 [96]
ET-1	T	ECs in normal liver [208]; epithelial TCs [117,208,209]; TECs [117,208], myofibroblasts, stromal cells [117,208]	↑ [117]; microvascular (+) associated with big ET-1 plasma level [209]	(+) [208]
	S		↑ versus C [117,208,209,232]; #, ↑ big ET-1 versus C [233]; big ET-1 not associated with OS or CSS [240]	↑ versus C [117,208]; ♦↑portal and systemic versus localized CRC [209]; ↑ in both LM types versus C [232]
ET AR, ET BR	T	TCs [114]	↑ ET AR mRNA, ↓ ET BR mRNA versus C [114]	
Galanin	T	submucosal and MPs cells, TCs, intestinal epithelium [67]	#, ↑ [120,121]	
	S		↑ 2.4x level versus C [67]	
G, G17, PG	T	cell membranes of TCs [210]; epithelial TCs [123,211]	♦, ↑ [47]; ↑ [123]; ♣, ↑ [210]; 39% (+) cells [211]	(+) [210]
	S		♦,↑ (G) in CRC with lymph node M versus CRC without M [234]; ↑ (G17) in CRC versus C [235]; ↑(G) not associated with any colonic neoplasms [241]	
GCG	T	TCs [210]; L cells [213]; EG cells [214]	↑ [210]; (+) [213]	
GLP1/2	T	human L cells [213];TCs of NET [236]	(+) [213,236]	
	S		↑/↑ [236]	
GLP2R	T	EECs in normal mucosa (GLP2R), cytoplasm of TCs [215]	(+) 20% CRC; 0% in polyps [215]	
GRPR	T	Tumor stromal (95%) and epithelial TCs (15%) [211]	↑ versus C; 100% (+); BRS-3-ND [135]; ♦,#,↓ [142]; ♣, (+) [144]; 100% (+) [211]	↓ mRNA versus pCRC [135]; ♦, #, lack or ↓ [142]; ND [144]
NmB	T	TCs and normal epithelium [141]	↑ in all but one tumor samples [135]; (+) [141]	↓ in LM versus pCRC [135]
NmB-R	T	TCs and normal epithelium [141]	ND [135]; ↑ versus C; ♣, ↓ [136]	
NK-1R	T	Peritumoral host vein cells [194]; TCs [218]	3-5-fold ↑ [194]; ♦,#, ↑ versus C [218]	
NT	S		↑3.7x in colon pathology versus C [49]; ↑ versus C and GC [237]; ↑ versus C [238]	
NTSR1	T	Epithelial (35%) and mesenchymal TCs (12%) [211]; colonic epithelial cells [227,229]	(+) 50% [211]; *NS* in colitis-CRC versus sporadic CRC [227]; ♣, moderate to strong expression [229]	
Pro-GCG, Glicentin	T	L cells [213]	(+) [213]	
PYY, PP/proPP-like peptides	T	human L cells [213]; TCs of NET [236]	(+) [213,236]	
	S		↑PYY [236]; PYY *NS* versus C, ↓PYY versus GC [237]	
Serotonin	T	EECs [214]	(+) [214]	
	S		♦, #, ↑ versusC and polyps [231]	
5HT3, 5HT4	T		(+) [106]	
SM	T	D cells [214,221]; TCs [221]	♣, ↓ [210]; ↓ [221]	
Sst1-sst5	T	TCs [222,223,230]	(+) SSt2 mRNA (20-50% CRC), ND in stage D, and LM [222]; ♣ (lower grade)-↑ Sst2, ♦, #, ↓ Sst2 and sst5 [223]; #, ↑ sst2 mRNA [230]	Sst2 mRNA ND in LM [222]; Sst2 and sst5-negative correlation with LM [223]
SP	T		♦,#, ↑ versus C [218]	
	S		↑ levels versus C [239]	
TrK	T	TCs of pCRC and peritoneal metastases [183]	↓ 10-fold TrKC in 60% CRC [178]; #, ♦, ↑ BDNF, #,↑ TrKB; ♦, #, ↑ TrKB [181]; ♦, (+) 23.3% (TrKB) and 12.8% (TrKC) [182]; ♦, #, ↑ BDNF/TrKB [183]	↑TrKB related to distant M [181]; TrKC related to LM [182]; BDNF alone, and BDNF+TrKB associated with LM [183]
VIP	T	cell membranes of signet-ring cells [212]; EECs [214]	(+) [212,214]	
	S		*NS* versus C, ↑ versus GC [237]	↑ versus C [158]
VPAC1	T	TCs, blood vessels near CRC, TAMs [95]; mucosal cells [216]	♣, ↑ VPAC1 [95]; (+) 96% CRC [216]	

Legend: ACE—Angiotensin I Converting Enzyme; AT1R/2R—Angiotensin 1/2 receptors; BBS—Bombesin; BRS-3—Bombesin Receptor subtype 3; C—controls; CCK-BR (CCK2R)—Cholecystokinin-B/gastrin receptor; CRC—colorectal carcinoma; CRHR1/CRHR2—Corticotropin-releasing Hormone (Factor) Receptors; CSS—Cancer-Specific Survival; DFS—Disease-Free Survival; ECs—Endothelial Cells; EECs—Enteroendocrine Cells; ET/AR/BR—Endothelin/Receptor A/B; GC—Gastric Cancer; GCG—Glucagon; GLP1/2/R—Glucagon-Like Peptides/Receptor; GRPR—Gastrin-Releasing Peptide Receptor; 5HT3/4—5HT (Serotonin) Receptors; M—metastases; mCRC—metastatic CRC; n—number of cases; ND—not detectable; NET—Neuroendocrine Tumor; NK-1R—Neurokinin 1 Receptor; NmB/R—Neuromedin B/Receptor; NS—non significant; NT/NTSR1—Neurotensin/NT Receptor 1; OS—Overall Survival; pCRC—primary CRC; PG—progastrin; PP—Pancreatic Peptide; PYY—Peptide YY; SM—Somatostatin; SP—Substance P; sst—Somatostatin Receptors; TAC1—tachykinin-1; TAMs—Tumor-Associated Macrophages; TCs—Tumor Cells; TECs—Tumor Endothelial Cells; TrK—Tropomyosin-related Kinase; VIP—Vasoactive Intestinal Peptide; VPAC1—VIP Receptor 1; (+)—positive expression; ↑/↓—significantly increased/decreased T expression/S level as related to C; ♣—significant association between NPs expression and grade (cancer differentiation); ♦—association between NPs expression and more advanced clinical stage of cancer (TNM, tumor size, venous infiltration, microsatellite nodules, metastases, etc.); #—significant correlation with poor prognosis (OS, DFS, CSS, etc.).

**Table 3:** Neuropeptides (NPs) and their receptors (NP-Rs) as potential targets and/or biomarkers during the treatment of CRC liver metastases.

NPs/NP-R	Model of Research	Type of Treatment	Therapeutical Effects	Ref.
SM receptor (SSR, Sst)	SSR (Sst2)-transfected CC531 CRC cells in a rat LM model (CC2B LM)	PRRT; 185 or 370 MBq (177 Lu-DOTA0, Tyr3) octreotate	significant antitumor response in rats with CC2B LM (SSR+) versus controls	[276]
SM analog (octreotide), galanin, serotonin	SW 620 CRC cells, nude (C57BL/6JBom-nu) mice	octreotide, galanin and serotonin	↓ incidence of metastases to the peritoneal cavity in the treated animals ((but *NS*); ↑ LM and to the intra-abdominal lymph nodes in controls; ↓ tumor volume, wet weight, proliferation index and number of tumor blood vessels in the treated animals	[277]
RAS	mouse CRC cells (MoCR); dimethylhydrazine-induced CRC in a CBA mouse with LM	rbesartan (AT1R blocker), captopril (ACE blocker), CGP42112A (AT2R agonist), and/or ANG-(1-7)	failed to show any benefit of combined targeting of the RAS	[278]
	diabetic mouse model, LM of CT26 mouse CRC cells	anti-ANG treatment with a chymase inhibitor, a renin inhibitor, and an ANG II receptor blocker	concurrent hypoglycemic and anti-ANG treatments showed a synergistic inhibitory effect on CT26 cell liver metastasis	[279]
	orthotopic murine model of CRC LM	ANG II, ANG-(1-7), captopril	↑ KC numbers in the liver but not tumor; captopril reduced growth of LM	[225]
NPY	N = 100 of metastatic CRC patients; ctDNA with mNPY	Regorafenib as last-line treatment	#, ↑ baseline level of ctDNA of mNPY	[280]

Legend: ANG—angiotensin; ACE—ANG converting enzyme; AT1R/2R—ANG II type 1/2 receptor; CRC—colorectal cancer; ct—circulating tumor; KC—Kupffer cells; LM—liver metastases; m—methylated; n—numer of patients; NPY—Neuropeptide Y; NS—non significant; PRRT—Peptide Receptor Radionuclide Therapy; RAS—Renin Angiotensin System; SM—somatostatin; Ref.—number of references; ↑/↓—significantly increased/decreased; #—significant correlation with poor prognosis (Overall Survival).

## Abbreviations

aa	amino acids
*APUD*	Amine Precursor Uptake and Decarboxylation
CI	Confidence Interval
ACE	Angiotensin I Converting Enzyme
AKT	Serine-threonine Protein Kinase (now called AKT1)
AT1R/2R	Angiotensin 1/2 receptors
BBS	Bombesin
CCK-BR (CCK2R)	Cholecystokinin-B/gastrin Receptor
c-Met	Tyrosine-protein Kinase Met or Hepatocyte Growth Factor Receptor
CN	Cranial Nerve
CRC	Colorectal Carcinoma
CRHR1/CRHR2	Corticotropin-releasing Hormone (Factor) Receptors
CSS	Cancer-Specific Survival
DFS	Disease-free Survival
ECs	Endothelial Cells
ECM	Extracellular Matrix
EECs	Enteroendocrine cells
EGF/R	Epidermal Growth Factor/Receptor
EMT	Epithelial-mesenchymal Transition
ERK1/2	Extracellular Signal-regulated Kinase 1/2
ET/AR/BR	Endothelin/Receptor A/B
FAK	Focal Adhesion Kinase
GC	Gastric Cancer
GCG	Glucagon
GLP1/2/R	Glucagon-Like Peptides/Receptor
GPCR	G Protein-coupled Receptors
GPR56	G Protein-coupled Receptor 56
GRP/R	Gastrin-Releasing Peptide/Receptor
HB-EGF	Heparin-binding EGF-like Growth Factor
HR	Hazard Ratio
HSCs	Hepatic Stellate Cells
5HT3/4 –5HT	Serotonin Receptors
IL	Interleukin
LM	Liver Metastasis
MAPK	Mitogen-activated Protein Kinase (originally called ERK)
MMP2, -7, -9	Matrix mmetalloproteinase 2, -7, -9
NET	Neuroendocrine Tumor
NF-κB	Nuclear factor kappa-light-chain-enhancer of activated B cells
NK-1R	Neurokinin1 Receptor
NmB/R	Neuromedin B/Receptor
NT/NTSR1	Neurotensin/NT Receptor 1
OR	Odds Ratio
OS	Overall Survival
PFS	Progression-free Survival
PI3	Phosphatidylinositol 3-kinase
PKA, B (AKT), C	Protein Kinase A, B (AKT), C
PG	Progastrin
PP	Pancreatic Peptide
PYY	Peptide YY
RFS	Recurrence-free Survival
SM	Somatostatin
SP	Substance P
Sst	Somatostatin Receptors
TAC1	Tachykinin-1
TAMs	Tumor-Associated Macrophages
TECs	Tumor Endothelial Cells
TGF-α, β	Transforming Growth Factor α, β
TNF-α	Tumor Necrosis Factor α
TNM	T—primary tumor, N—regional lymph nodes, M—distant metastasis
TrK	Tropomyosin-related Kinase
VEGF	Vascular Endothelial Growth Factor
VIP	Vasoactive Intestinal Peptide
VPAC1	VIP Receptor 1
